# Editorial: Root development: Towards understanding regulatory networks and complex interactions between cell populations

**DOI:** 10.3389/fpls.2022.1108367

**Published:** 2023-01-06

**Authors:** Svetlana Shishkova, Ling Huang, Ramiro Esteban Rodríguez, Daniela Ristova, Raffaele Dello Ioio

**Affiliations:** ^1^ Departamento de Biologa Molecular de Plantas, Instituto de Biotecnología, Universidad Nacional Autónoma de México, Cuernavaca, Mexico; ^2^ Integrative Genomics and Bioinformatics Core, Salk Institute for Biological Studies, La Jolla, CA, United States; ^3^ Instituto de Biología Molecular y Celular de Rosario (IBR) - National Scientific and Technical Research Council and Universidad Nacional de Rosario, Rosario, Argentina; ^4^ Institute for Plant Sciences, Cologne Biocenter, University of Cologne, Cologne, Germany; ^5^ Dipartimento di Biologia e Biotecnologie, Sapienza Università di Roma, Rome, Italy

**Keywords:** root growth, cell proliferation and differentiation, root plasticity, root system architecture, gene regulatory networks

The root system architecture is pivotal for soil exploration and plant adaptation and survival. For this reason, the genetic control of root development (reviewed, e.g., in Bennett and Scheres, 2010; Slovak et al., 2016) is under enormous selection pressure at various scales, from tissue patterning to branching of the below-ground root system, which can be even more extensive than the above-ground shoot system.

Various aspects of root development are discussed in this collection, including the role of hormonal cross-talk in root development in general, as well as auxin, peptide hormones, ROS homeostasis and cell-wall proteins, in particular. Also, traits of the root system architecture and differences in ground tissue patterning between species are discussed, among other subjects.

Plant hormones are main protagonists in the control of root development. In this special issue Zluhan-Martínez et al. and García-Gómez et al. underpin their role in root patterning and growth, shedding light on the cross-talk among several hormones and cell proliferation and patterning. While García-Gómez et al. highlight how hormones interact with master regulators of stem cell activity to maintain stem cell identity, Zluhan-Martínez et al. report the most recent findings on cell proliferation and differentiation.

The phytohormone auxin influences root development in multiple ways and at many levels, being a signal that induces drastic changes in gene expression. The AUXIN RESPONSE FACTOR (ARF) family proteins are the transcription factors at the end of the auxin signaling cascade. Kirolinko et al. report on the participation of the ARF2, ARF3 and ARF4 from *Medicago truncatula* in lateral root and nitrogen-fixing nodule development, thus expanding our knowledge of the participation of the miR390-tasiARF-ARF regulatory node to this model legume species.

Plant peptide hormones participate in signaling cascades by binding to specific membrane receptor kinases. Like traditional plant hormones, later-described peptide hormones also have diverse regulatory roles in plant development and physiology. Hussain et al. identify that a peptide hormone family member *PAMP-INDUCED PEPTIDE 2* (*PIP2*) in *Arabidopsis thaliana* regulates both root and hypocotyl elongation. As *PIP2* is an auxin responsive gene, it provides another remarkable example on how cross-talk between traditional plant hormones and plant peptide hormones collectively regulates plant growth and development.

Besides traditional plant hormones and peptide hormones, other compounds act as internal and external cues to regulate plant root development and mediate its response to environmental changes.

Reactive Oxygen Species (ROS) were initially conceived as dangerous by-products of oxygen metabolism in aerobic organisms, while by now their role in development and signaling pathways is clearly established. In this Research Topic, Mase and Tsukagoshi review the role of ROS homeostasis in root development and integrate their signaling role with plant hormones and transcription factors. The authors provide an extensive description of the multiple aspects of root development modulated by ROS, among them the promotion of the polar tip growth of the root hairs. In line with this, Kim et al. identify and characterize in rice a regulatory module composed of a Rho-type GTPase of Plants (ROP/Rac) that interacts with a particular ROP-guanine nucleotide exchange factor and a respiratory burst oxidase to promote root growth in rice.

Transcription factors from plant-specific AP2/ERF superfamily play essential roles in many aspects of plant development and stress response. Wang et al. demonstrate that the overexpression of *PagERF16* in *Populus alba* × *P. glandulosa* hybrids results in an increase in root diameter and volume. On the other hand, *PagERF16* overexpression lines were sensitive to salt stress, showing a decrease in the total root length in comparison with WT hybrid lines.

Although representing a minor proportion of the plant cell wall constituents, structural proteins are essential components. These include proline rich proteins, glycine-rich proteins, extensins and arabinogalactan proteins (AGP), the latter being glycoproteins with galactose and arabinose as the most abundant sugar moieties. Hromadová et al. review in this Research Topic the multiple roles of cell-wall localized AGPs in root development, stress response and in mediating the interaction with other organisms.

Plants show a large interspecific diversity in root radial patterning (Di Ruocco et al., 2018). *Arabidopsis thaliana* ground tissue patterning has been widely used to understand the molecular basis of radial patterning in roots. In their review Hernandez-Coronado and Ortiz-Ramirez first describe the molecular mechanisms governing radial patterning of the ground tissue in the *A. thaliana* root meristem. Subsequently, they highlight how these findings allowed the comprehension of the molecular basis of root radial patterning diversity among different plant species.

The root system has crucial importance for plant development and fitness, yet the root traits were rarely part of plant breeding strategies. Deja-Muylle et al. report a comprehensive study of 17 root system architecture (RSA) traits in 241 *Arabidopsis thaliana* accessions grown in large plates, as well as in rhizotrons. They identified an overall correlation of *in vitro* RSA traits and RSA traits of plants grown in soil, but not for all accessions, suggesting that later stages of root development can be shaped uniquely by the environment. Additionally, the authors report many known and newly identified genome-wide associations for 14 root traits.

Root system architecture is also a focus of González-Sánchez et al. study. They recorded the dynamics of RSA of closely related and more divergent species from a large genus *Mammillaria* belonging to the Cactaceae family. Determinate growth of cacti primary and lateral roots (Shishkova et al., 2013) allowed to follow root growth in 12 cm square petri plates during more than five months after seed germination. The authors conclude that the phenotypic outcome of microevolution of *Mammillaria* RSA partially recapitulates the patterns generated at the macroevolutionary level in this genus.

Development of fully functional root system has evolutionary significance that enabled plants to colonize lands. Fang et al. in their review, focus on the importance of the evolution of lycophyte roots (*Selaginella*) with an emphasis on root apical meristem (RAM) organization, root branching, and auxin control of root development. Moreover, exploiting genomics and transcriptomics knowledge the authors stress the importance of auxin homeostasis in *Selaginella* root development, and pin-point developmental genes and protein families that play crucial role in lycophytes evolution.

Single-cell RNA-sequencing (scRNA-seq) has been shown to be a powerful tool to profile transcriptional signatures at unprecedented resolution to unravel cell identity and reconstruct gene regulatory networks (GRN) (Minne et al., 2022). Serrano-Ron et al. summarize different strategies used for scRNA-seq and demonstrates how it can be useful to understand the molecular mechanism of lateral root formation, a field that remains largely unexplored by the current knowledge.

In conclusion, the thirteen articles in this collection highlight multiple features of root development from the molecular and cellular level to the whole root-system level in model and non-model plant species.

## Author contributions

All authors of the Editorial wrote and approved it for publication and contributed to the Research Topic.
